# Diagnostic value of plasma SIRT1 levels and whole-brain gray matter volume in Parkinson’s disease patients with cognitive impairment

**DOI:** 10.1007/s10072-023-07071-6

**Published:** 2023-09-18

**Authors:** Xiaohuan Li, Dawei Yang, Jianjun Ma, Wei Wei, Jinhua Zheng, Yongyan Fan, Keke Liang, Xiaoxue Shi, Dongsheng Li, Zonghan She, Xuelin Qi, Siyuan Chen

**Affiliations:** 1 Department of Neurology, Zhengzhou University People’s Hospital, Zhengzhou, China; 2 Department of Neurology, Henan Provincial People’s Hospital, Zhengzhou, China; 3 Department of Neurology, Henan University People’s Hospital, Zhengzhou, China

**Keywords:** Parkinson’s disease, SIRT1, Cognitive impairment, Whole-brain gray matter volume

## Abstract

**Objective:**

This study was designed to investigate the diagnostic value of plasma SIRT1 levels and whole-brain gray matter (GM) volume in Parkinson’s disease (PD) patients with cognitive impairment.

**Methods:**

Automated enzymatic analysis was performed to measure plasma SIRT1 levels in 80 healthy controls and 77 PD patients. Motor symptoms and nonmotor symptoms in PD patients were assessed using the corresponding scales. A Siemens MAGNETOM Prisma 3 T MRI scanner was used to acquire images in 35 of 77 PD patients.

**Results:**

Plasma SIRT1 levels in PD patients were lower than those in healthy controls. Plasma SIRT1 levels were negatively correlated with the age, Unified Parkinson’s Disease Rating Scale Part III (UPDRS-III) scores, anxiety, depression, excessive daytime sleepiness (EDS), quality of life, and especially cognitive impairment. Thus, it showed that plasma SIRT1 levels were relevant to visuospatial/executive function, memory, and language. Receiver-operating characteristic (ROC) analysis confirmed that plasma SIRT1 levels had good diagnostic accuracy for PD with anxiety and EDS. Furthermore, plasma SIRT1 levels had a significant positive correlation with GM volume in the whole brain, and ROC analysis confirmed that plasma SIRT1 levels and the total GM volume had good diagnostic accuracy for PD with cognitive impairment.

**Conclusions:**

This study showed that plasma SIRT1 levels were correlated with the nonmotor symptoms of anxiety, depression, EDS, and especially cognitive impairment as well as the total GM volume. Furthermore, the combination of plasma SIRT1 levels and the total GM volume had good diagnostic accuracy for PD with cognitive impairment.

## Introduction

Parkinson’s disease (PD) is a common clinical neurodegenerative disease that is mainly related to the degeneration and death of dopaminergic neurons in the substantia nigra of the midbrain [1]. The onset of PD is insidious and its progression is slow. PD not only features motor dysfunction, such as bradykinesia, static tremor, and myotonia, but also frequently comes accompanied by nonmotor symptoms, such as cognitive impairment, depression, anxiety, sleep disorders, and sensory abnormality [2]. Cognitive impairment is one of the main nonmotor symptoms [3]. Of all patients newly diagnosed with PD, approximately 1/3 will develop cognitive impairment. Approximately 26% of PD patients develop Parkinson’s disease dementia (PDD) 5 years after diagnosis, and up to half of the rest have mild cognitive impairment (MCI) [4]. Early diagnosis and early treatment can improve the social function and quality of life of PD patients with cognitive impairment [5].

SIRTs are nicotinamide adenine dinucleotide (NAD +)-dependent histone deacetylases, and SIRT1 is one of the most widely studied members of the SIRT family [6]. Some experimental studies have found that SIRT1 is related to the pathogenesis of PD [7]. In animal and cell models of PD, SIRT1 can inhibit the aggregation of α-Syn by activating molecular chaperones, and deacetylase activity mediates the clearance of α-Syn by autophagy mediated by light chain 3 (Lc3) [8]. SIRT1 may also increase the degradation of α-Syn oligomers by deacetylating HSF1, upregulating HSP70 transcription, and increasing the degradation of α-Syn oligomers [9]. In addition, SIRT1 is involved in the regulation of oxidative stress, inflammatory response, and mitochondrial function in PD [7]. At the same time, some studies have shown that SIRT1 has a neuroprotective effect on PD by deacetylating histones and many transcription factors, such as p53 and PGC-1α, to resist the neurotoxicity of α-Syn [10]. There is compelling evidence to support the fact that SIRT1 is shuttled between the nucleus and cytoplasm and perform context-dependent functions in neurodegenerative diseases including Alzheimer’s disease, Parkinson’s disease, and Huntington’s disease [6]. Besides, recent study found that SIRT1 is relevant to cognitive impairment in PD [11].

Voxel-based morphometry (VBM) is a commonly used method of brain structure analysis based on MRI. It can objectively evaluate the early morphological changes in brain structure in vivo, is especially sensitive to the early morphological changes of neurodegenerative diseases, and can provide important information for early diagnosis [12]. VBM has been widely used to analyze the differences in brain structure in different populations, among which there are many studies on the brain structure of PD cognitive impairment. There is extensive structural atrophy of the cerebral cortex in PD patients with cognitive impairment, including the frontal lobe, temporal lobe, parietal lobe, occipital lobe, limbic system, insular lobe, and hippocampus [13]. In addition, previous studies showed that gray matter (GM) changes in PD are relevant to cognition [14].

At present, there is no specific diagnostic index for PD with cognitive impairment. Some indexes are biomarkers such as SIRT1; some are neuroimages. We aim to combine the two. In our study, we not only measured plasma SIRT1 levels but also total GM volume using VBM. The purpose of this study was to explore the diagnostic value of plasma SIRT1 levels combined with total GM volume through receiver-operating characteristic (ROC) analysis in PD patients with cognitive impairment to find a better diagnostic index for PD patients with cognitive impairment.

## Materials and methods

### Participants

Seventy-seven patients with clinically diagnosed PD were recruited continuously from 2021 to 2022. The patients were diagnosed by two experienced neurologists according to the UK PD Society Brain Bank Clinical Diagnostic Criteria for PD [15]. Patients with Parkinson’s syndrome or secondary PD caused by cerebrovascular disease, poisoning, encephalitis, trauma, or drugs, as well as patients who had PD complicated with AD, were excluded. A total of 80 healthy volunteers participated in the study. Exclusion criteria were as follows: (1) patients with severe cognitive impairment such as Alzheimer’s disease, Lewy body dementia, and vascular dementia; (2) patients with Parkinson’s superposition syndrome such as multiple system atrophy and progressive supranuclear paralysis, as well as patients with secondary Parkinson’s syndrome, such as drugs and toxic vessels; (3) patients after tumor or deep brain stimulation; (4) patients with hypertension, diabetes, or severe heart, liver, lung, and kidney dysfunction.

### Clinical data

The general data of selected PD patients such as age, sex, disease duration, and levodopa equivalent daily dose (LEDD) were recorded and counted. Motor symptoms were assessed using Part III of the Unified Parkinson’s Disease Rating Scale (UPDRS). Nonmotor symptoms were assessed by the REM Sleep Behavior Disorder Questionnaire–Hong Kong edition (RBDQ-HK), Pittsburgh Sleep Quality Index (PSQI), Parkinson’s Disease Sleep Scale (PDSS), Epworth Sleepiness Scale (ESS), Non-Motor Symptom Scale (NMSS), Hamilton Anxiety Scale (HAMA-14), Hamilton Depression Scale (HAMD-17), and Montreal Cognitive Assessment (MoCA). The severity of the disease was evaluated by the modified Hoehn and Yahr (H-Y) scale and the UPDRS. The quality of life of the patients was evaluated with the 39-item PD Questionnaire (PDQ-39). All of the assessments were completed once during a patient’s “on” period. Medical history inquiry, physical examination, and scale scoring were carried out by trained neurologists.

### Blood sample collection

Peripheral blood was collected in test tubes without anticoagulant from each subject from 07:30 to 08:30 AM after an overnight fast and before breakfast. The sample was allowed to coagulate at room temperature for 30 min, the blood was centrifuged (1000 × *g*, 15 min), and the serum was removed and stored at − 80 °C until measurement. All of the samples were collected during patients’ “on” period.

### Measurement of plasma SIRT1 levels

Plasma SIRT1 levels were measured using an enzyme-linked immunosorbent assay (ELISA) kit (MyBiosource, USA). The detection range of this kit is 0.31–20 ng/ml, the sensitivity is 0.19 ng/ml, and the intra and inter detection variability ranges are ≤ 8% and ≤ 12%, respectively. Biological replicates were analyzed on the same plate according to the manufacturer’s instructions.

### MRI acquisition

Image acquisition was performed using a Siemens MAGNETOM Prisma 3 T MRI scanner with a 64-channel head coil with the following parameters for the T1-weighted 3D-MPRAGE sequence: echo time (TE) = 3.43 ms, repetition time (TR) = 5000 ms, inversion time (T1) = 755 ms, flip angle = 4°, slice thickness = 1.00 mm, slice number = 208, bandwidth = 240 Hz/pixel, a matrix of 256 × 256, field of view = 256 × 256 mm^2^, and voxel size = 1.0 × 1.0 × 1.0 mm^3^. All of the images were collected during patients’ “on” periods, and images of 35 of 77 PD patients were acquired.

### Voxel‑based morphometry analysis

The Gaussian default longitudinal preprocessing approach in the VBM8 toolbox was used with the following standardized steps: (1) registering the follow-up image to the baseline image for each subject; (2) calculating the mean image from the realigned images for each subject and using it as a reference image for subsequent spatial realignment; (3) correcting the realigned images for signal inhomogeneities with regard to the reference mean image; (4) performing tissue segmentation in the bias-corrected mean reference image and the bias-corrected realigned images; (5) estimating Diffeomorphic Anatomical Registration Through Exponentiated Lie Algebra (DARTEL) spatial normalization parameters with the tissue segments of the bias-corrected mean reference image; (6) modulating GM images to preserve relative regional volumes and correct for individual differences in brain size; (7) applying normalization parameters to the tissue segments of the bias-corrected realigned images; and (8) smoothing the resulting normalized tissue segments for each time point of each subject with an 8-mm full-width-half maximum (FWHM) Gaussian kernel [16]. Thirty-five patients were assessed only at baseline.

### Statistical analysis

SPSS26.0 software was used for statistical analysis. Quantitative data that followed the normal distribution according to the Kolmogorov–Smirnov test were expressed as the mean ± standard deviation, and the comparison between the two groups was performed by two independent sample *t* test. Data that did not conform to the normal distribution were represented as the median (quartile range) and were compared using the Mann–Whitney *U* test. Enumeration data were expressed as the percentage of cases, and the chi-square test was used for comparison. The diagnostic value of plasma SIRT1 level and the total GM volume for PD with cognitive impairment were evaluated by ROC curve analysis. Spearman correlation analysis was used to evaluate the correlation between plasma SIRT1 levels and each index as well as the total GM volume. All tests were double-tailed, and a probability (*P*) value less than 0.05 was considered statistically significant.

## Results

### Demographic data and plasma SIRT1 levels of normal subjects and patients with PD

**Fig. 1:**
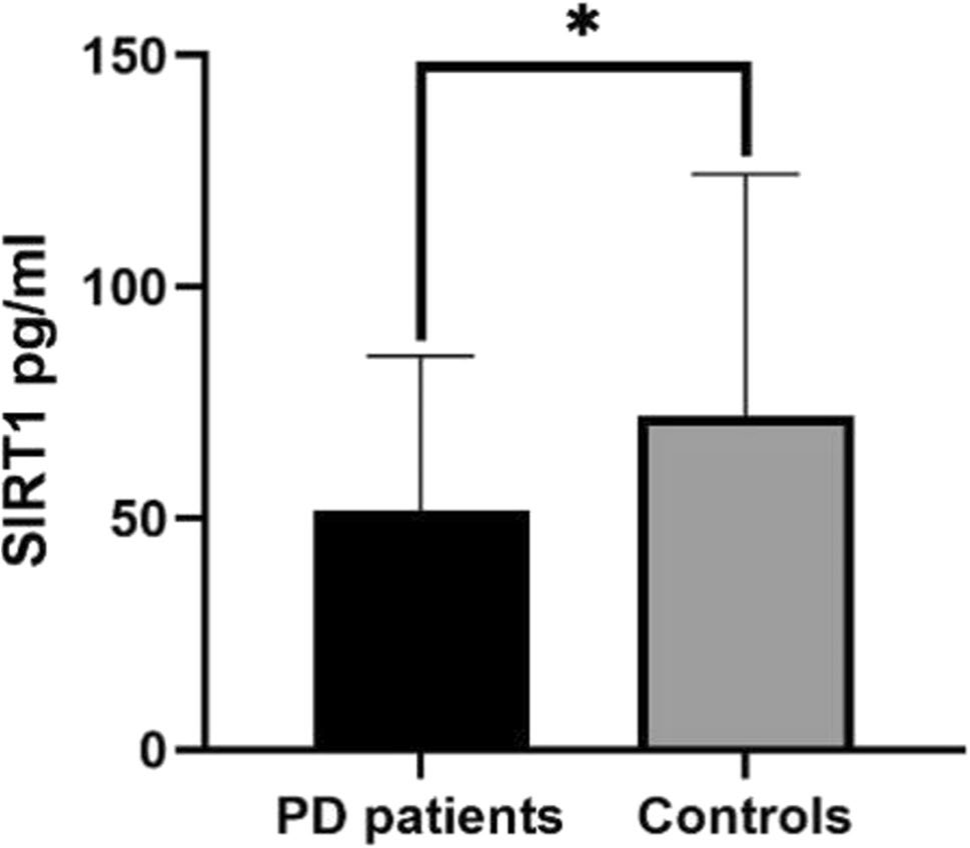
Differences in plasma SIRT1 levels between PD patients (*n* = 77) and controls (*n* = 80). *P < 0.05, **P < 0.01, ***P < 0.001

There was no significant difference in age or sex between the PD group and the control group (65 (57, 68) vs. 60 (56, 65), *P* = 0.068; 55.55% vs. 53.75%, *P* = 0.92), but the plasma SIRT1 level in the PD group was significantly lower than that in the normal control group (*P* = 0.025) (Fig. 1).

### Correlation between plasma SIRT1 levels and clinical data or nonmotor symptoms in patients with PD

**Fig. 2:**
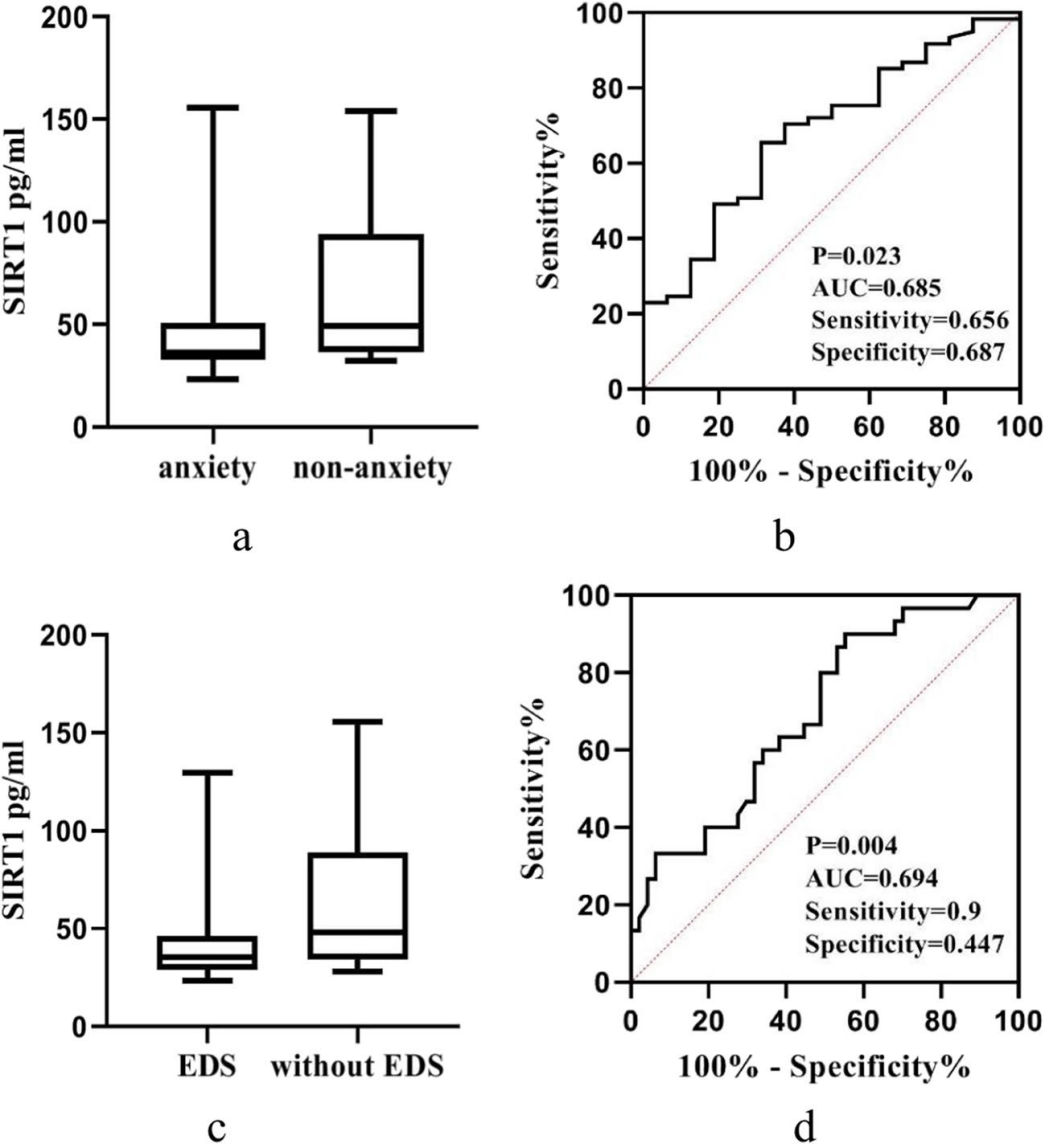
**a** Plasma SIRT1 levels in the anxiety and non-anxiety subgroups of PD patients. PD patients with anxiety showed lower levels of plasma SIRT1. **b** Identification of PD with anxiety based on plasma SIRT1 levels determined by ROC analysis. **c** Plasma SIRT1 levels in the EDS and without EDS subgroups of PD patients. PD patients with EDS showed lower levels of plasma SIRT1. **d** Identification of PD with EDS based on plasma SIRT1 levels determined by ROC analysis

There was no correlation between SIRT1 levels and LEDD (Table 1). SIRT1 levels were negatively correlated with age, UPDRS-III scores, HAMD-17, HAMA-14, Epworth, and PDQ-39 scores but positively correlated with MoCA scores (Table 1). In other scales, there was no correlation between SIRT1 levels and scores (Table 1). Next, we used significantly related nonmotor symptoms as the grouping criteria for PD patients and compared the difference in SIRT1 concentration between each pair of subgroups. The subjects included 77 patients with Parkinson’s disease, with HAMD-17 ≥ 17 (representing depression) in 22 cases (28.6%), HAMA-14 ≥ 14 (representing anxiety) in 61 cases (79.2%), MoCA < 21 (representing cognitive impairment) in 38 cases (49.4%), and ESS ≥ 10 (representing EDS) in 30 cases (39.0%). There was no significant difference between the depression subgroup and the control subgroup in PD patients. Plasma SIRT1 levels in the anxiety subgroup and the EDS subgroup were lower than those in the non-anxiety subgroup and the without EDS subgroup, while plasma SIRT1 levels in the cognitive impairment subgroup were significantly lower than those in the non-cognitive impairment subgroup (35.71 (33.04, 49.58) vs. 38.57(33.26, 56.13), *P* = 0.289; 36.29 (32.83, 50.72) vs. 49.14 (36.23, 94.24), *P* = 0.023; 35.54 (29.91, 40.17) vs. 43.32(34.37, 65.25), *P* = 0.004; 34.21 (29.91, 46.30) vs. 42.62(35.81, 68.82), *P* = 0.001). According to the ROC curve analysis, the area under the curve (AUC) values of PD with anxiety and EDS based on plasma SIRT1 levels were 0.685 and 0.694, the sensitivity values were 65.6% and 90%, the specificity values were 68.7% and 44.7%, and the cutoff values were 39.20 and 49.13, respectively (Fig. 2a, b, c, d). The correlation analysis between SIRT1 level and MoCA subitem score showed that SIRT1 levels were significantly correlated with visuospatial/executive function, memory, and language scores (Table 2).

**Table 1:** Relationship between plasma SIRT1 levels and demographic or clinical data in patients with PD

	Means ± standard deviations/medians (quartile ranges)	Pearson/Spearman rank	*P* values
Age (y)	65 (57, 68)	− 0.240*	0.035
Age of onset (y)	60 (52, 65)	− 0.224	0.050
Disease duration (y)	3 (2, 5)	0.037	0.751
LEDD (mg/d)	395 (307.5, 587.5)	− 0.010	0.930
UPDRS-III score	30 (18, 38)	− 0.302**	0.008
HAMD-17 score	14 (10, 17)	− 0.237*	0.038
HAMA-14 score	17.58 ± 5.99	− 0.236*	0.039
MoCA score	21 (16.23, 50)	0.351**	0.002
RBDQ-HK score	14 (0, 32.50)	− 0.061	0.598
PSQI score	8 (5, 11.50)	− 0.089	0.439
PDSS score	123 (96.50, 133.50)	0.135	0.241
ESS score	6 (3, 14)	− 0.296**	0.009
NMSS score	45 (30.50, 62.50)	− 0.179	0.119
PDQ-39 score	37.27 ± 24.82	− 0.247*	0.031

**Table 2:** Relationship between plasma SIRT1 levels and each part scores of MoCA in patients with PD

MoCA subscore	Means ± standard deviations/medians (quartile ranges)	Pearson/Spearman rank	*P* values
Visuospatial/executive	2 (1, 3.50)	0.449*	0.000
Naming	3 (2, 3)	0.058	0.617
Memory	2 (1, 3)	0.434**	0.000
Attention	4 (3, 5)	0.197	0.086
Language	2 (2, 3)	0.370**	0.001
Abstractions	1 (0, 1.50)	− 0.140	0.226
Orientation	6 (4, 6)	0.092	0.424

### Correlation of plasma SIRT1 levels with whole-brain GM volume and their diagnostic values in PD patients with cognitive impairment

**Fig. 3:**
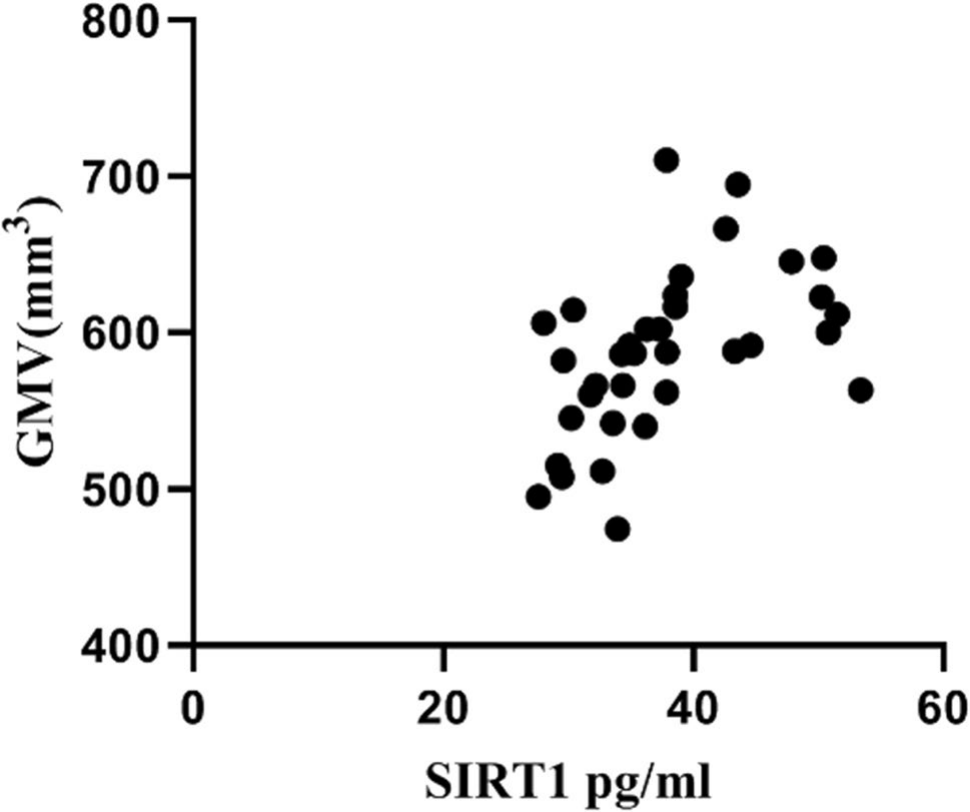
Relationship between plasma SIRT1 concentrations and whole-brain GM volume. Plasma SIRT1 levels were positively correlated with whole-brain GM volume (Pearson’s correlation coefficient *r* = 0.068, *P* < 0.001, *n* = 35)

**Fig. 4:**
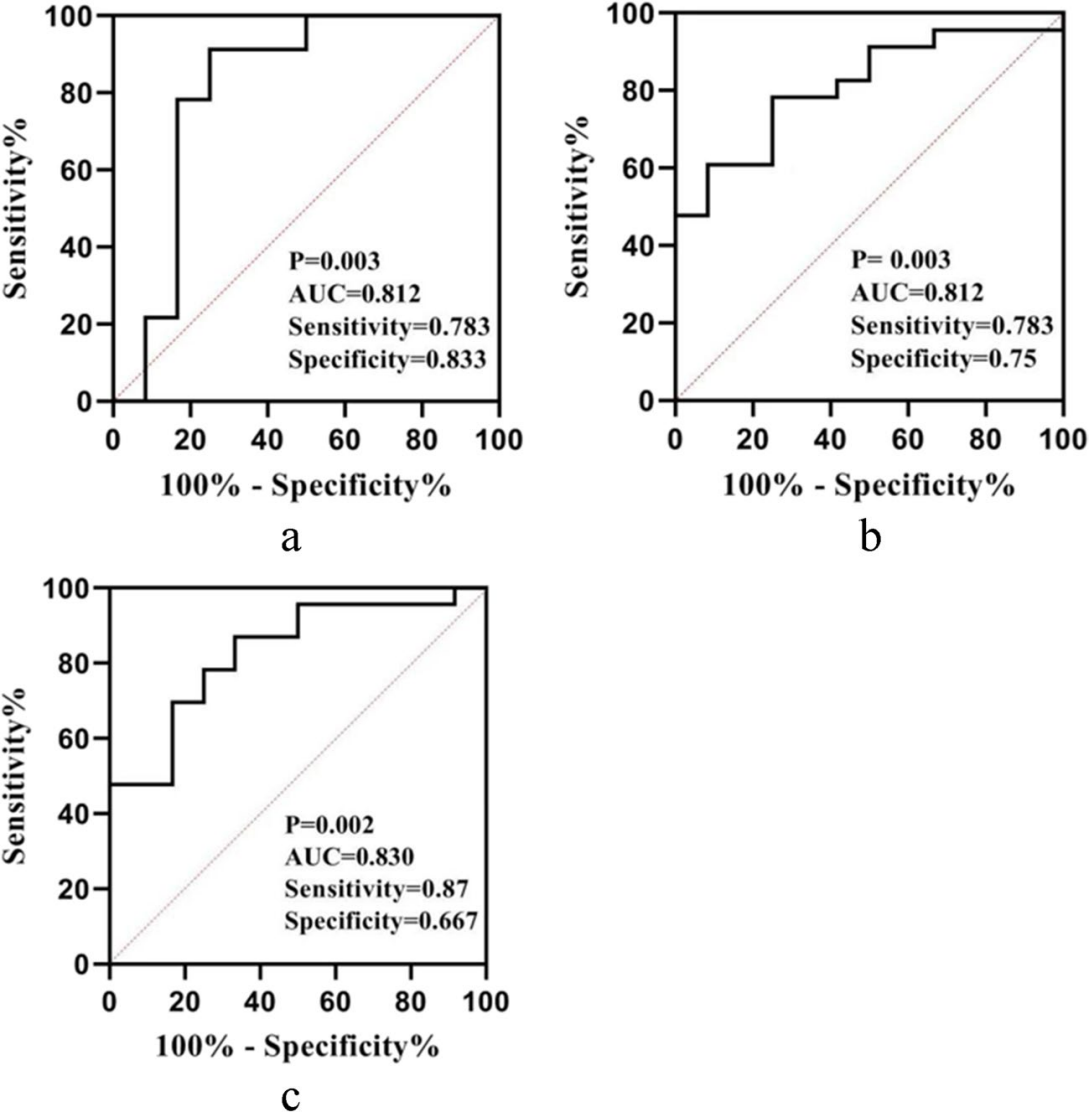
**a** Identification of PD with cognitive impairment based on plasma SIRT1 levels determined by ROC analysis. **b** Identification of PD with cognitive impairment based on total GM volume determined by ROC analysis. **c** Identification of PD with cognitive impairment based on plasma SIRT1 levels and whole-brain GM volume determined by ROC analysis

The level of plasma SIRT1 was significantly correlated with the volume of GM in the whole brain (*r* = 0.608**, *P* < 0.001, *n* = 35) (Fig. 3). According to the ROC curve analysis, the AUC values of PD with cognitive impairment based on plasma SIRT1 levels and whole-brain GM volume, respectively, were 0.812 and 0.812; the sensitivity values were 78.3% and 78.3%, and the specificity values were 83.3% and 75%. The optimal cutoff values were 35.09 and 584.12, respectively. The ROC curve for the combination of the two variables had an AUC value of 0.830, a sensitivity of 87%, and a specificity of 66.7%. Both the AUC value and the sensitivity were improved (Fig. 4a, b, c).

## Discussion

PD is the second most common age-related neurodegenerative disease [17]. The main pathological changes are (1) dysfunction and loss of dopaminergic neurons accompanied by oxidative stress, mitochondrial dysfunction and inflammation, or immune response and (2) the formation of Lewy bodies composed of α-Syn, heat shock protein, and ubiquitin [1]. Previous studies have shown that the loss of SIRT1 or its mutation in animal and cell models of PD may lead to PD pathology, and there is a genetic correlation between SIRT1 and PD [18]. At the same time, some studies found that SIRT1 mRNA and gene expression were downregulated in the peripheral blood of patients with PD [19]. Our study found that plasma SIRT1 levels decreased in patients with PD, consistent with previous studies showing that serum SIRT1 levels in patients with PD were lower than those in healthy controls [11]. Subsequently, we considered the factors that may affect the level of plasma SIRT1. Correlation analysis showed that the level of plasma SIRT1 was negatively correlated with disease severity. Previous studies have shown that α-Syn protein aggregation promotes mitochondrial dysfunction and reduces the expression of SIRT1 [18]. The more serious the disease is, the more α-Syn protein accumulates, which leads to a reduced level of SIRT1. Plasma SIRT1 is related to the progression of the disease. In previous PD model studies, SIRT1 resisted the neurotoxicity of α-Syn and produced neuroprotective effects in PD by deacetylating histones and many transcription factors, such as p53 and PGC-1α [10]. In addition, correlation analysis showed that plasma SIRT1 levels had a significant negative correlation with age. Previous studies have shown that the expression of SIRT1 decreases with age and participates in the regulation of cell senescence [20]. In addition, PD itself is an age-related neurodegenerative disease [17].

Nonmotor symptoms are characterized by cognitive, neuropsychiatric, autonomic, and sensory disorders, which usually worsen with the progression of the disease [21]. The evaluation and treatment of nonmotor symptoms may help to improve the health-related quality of life in patients with PD [2]. Correlation analysis showed that plasma SIRT1 levels were associated with nonmotor symptoms such as depression, anxiety, EDS, and cognitive impairment and negatively correlated with quality of life in patients with PD. Previous studies have shown that SIRT1 is involved in neuroinflammation and oxidative stress, and these two factors are closely related to emotional disorders [22]. In an animal model of PD, SIRT1 can inhibit the expression of inflammatory cytokines, including tumor necrosis factor-β (TNF-β), interleukin-1 (IL-1), and interleukin-6 (IL-6) [23]. PGC-1α is a multifunctional molecule that can activate many nuclear receptors and transcription factors [24]. PGC-1α can increase the activity of antioxidant enzymes and protein level to inhibit oxidative stress [25]. SIRT1 can deacetylate PGC-1α to maintain the high protein levels of this factor, thus enhancing its effect on antioxidant stress [7]. The decrease of SIRT1 levels in PD patients leads to the enhancement of inflammatory reactions and oxidative stress, which promotes the occurrence of depression and anxiety.

Previous studies have shown that patients with PD with EDS showed extensive cortical and subcortical changes, such as frontal lobe atrophy, combined cortical hypoperfusion, and decreased dopamine uptake by the dopamine transporter (DAT) in the caudate nucleus, and abnormal neural activity in patients with PD with EDS and PD without EDS, the abnormality of low frequency fluctuation amplitude (ALFF) at the local level in the pons and frontal lobe, the decrease of ALFF in cingulate cortex, and the reduction of functional connection at cingulate cortex and precuneus network level [26]. In the pathogenesis of PD, apoptosis is an important reason for the loss of dopaminergic neurons, resulting in a decrease in uptake by DAT. Studies have shown that SIRT1 can reduce the damage caused by neurodegenerative diseases by regulating the apoptosis of neurons [7]. Previous studies have confirmed that SIRT1 targets H3K9 histone and regulates the expression of the p53 gene at the transcriptional level, thus inhibiting the expression of the p53 gene to enhance neuroprotection [10]. Other studies have shown that SIRT1 deacetylates the C-terminal residues of the main ubiquitin site of p53 during cellular stress, which helps to prevent protein degradation and stabilize p53 [27]. The decrease in SIRT1 levels in PD patients leads to an increase in p53 gene expression and an increase in apoptosis of dopaminergic neurons and other neurons, thus promoting the production of EDS and abnormal neural activity.

The results of our study showed that there was a significant correlation between SIRT1 and PD with cognitive impairment, and further analysis showed that the level of SIRT1 was related to visuospatial/executive function, memory, and language. Previous studies have shown that serum SIRT1 is significantly decreased in patients with cognitive impairment and dementia. The worse their cognitive test scores were, the lower their serum SIRT1 levels, which is consistent with the results of this study [28]. The SIRT1 gene is mainly expressed in neurons and plays a key role in regulating the fate of neural progenitor cells, axonal dendritic differentiation, and synaptic plasticity [29]. In a mouse model, hippocampal SIRT1 gene knockout led to hippocampal atrophy in 8-month-old mice. Open field test and Morris Water Maze test showed that hippocampal SIRT1 gene knockout could significantly impair the spatial learning and memory ability of mice [30]. In addition, decreased SIRT1 levels can also increase the release of inflammatory mediators by microglia [31], which infiltrate into the white matter, causing damage and neuronal loss and resulting in learning and memory impairment [32]. Our study also demonstrated that SIRT1 is related to visuospatial/executive function and memory, which is consistent with the above results. To date, there have been few studies on the relationship between SIRT1 and language function, but in this study, the language disorder observed in patients with PD was mainly motor language disorder, which is driven by changes in the frontal lobe. SIRT1 may affect language function by regulating the apoptosis of frontal lobe neurons and neuroinflammation [10, 31].

Finally, we discussed the correlation between plasma SIRT1 levels and whole-brain GM volume and their diagnostic values in PD patients with cognitive impairment. We found that the level of plasma SIRT1 was positively correlated with whole-brain GM volume. We did not find a relationship between region-specific GM volumes in the brain and plasma SIRT1 levels, probably because we included too few patients in the imaging study. We considered the following reasons in patients with PD to reach this conclusion. Studies have shown that the level of plasma SIRT1 is negatively correlated with the severity of PD. At the same time, studies have confirmed that global GM loss, amygdala atrophy, and frontotemporal cortex thinning are particularly associated with the degenerative process of PD in patients with early PD [33]. The results of this study showed that there was a significant correlation between plasma SIRT1 and PD with cognitive impairment; furthermore, the whole-brain GM volume had diagnostic value for PD with cognitive impairment. Combining plasma SIRT1 levels with total GM volume into ROC analysis, it was found that both the AUC and the sensitivity increased.

This study has the following limitations. First, the evaluation of nonmotor symptoms may have been affected by subjective factors; cognitive impairment of PD patients was evaluated according to MoCA, which was able to define cognitive impairment in PD at the first level of accuracy only [34]; in future studies, we can adopt more objective evaluation methods. Second, we assessed patients only at baseline while using VBM in the cross-sectional study. In the presence of various confounding factors, it is best to conduct a longitudinal study to clarify the relationship between the occurrence of cognitive impairment in PD and total GM volume.

## Conclusion

This study showed that plasma SIRT1 levels were correlated with the nonmotor symptoms of anxiety, depression, EDS and especially cognitive impairment, along with whole-brain GM volume. The combination of plasma SIRT1 levels and total GM volume had good diagnostic accuracy for PD with cognitive impairment.

## Data Availability

Data will be made available on request from the corresponding authors.
